# Patterns and influencing factors of exercise attendance of breast cancer patients during neoadjuvant chemotherapy

**DOI:** 10.1007/s00520-023-08269-2

**Published:** 2024-01-03

**Authors:** Siri Goldschmidt, Martina E. Schmidt, Friederike Rosenberger, Joachim Wiskemann, Karen Steindorf

**Affiliations:** 1 Division of Physical Activity, Prevention and Cancer, German Cancer Research Center (DKFZ), and National Center for Tumor Diseases (NCT), Heidelberg, a partnership between DKFZ and University Medical Center, Heidelberg, Germany; 2 Medical Faculty of the University of Heidelberg, Heidelberg, Germany; 3 Department of Medical Oncology, University Medical Center, and National Center for Tumor Diseases (NCT), Heidelberg, a partnership between DKFZ and University Medical Center, Heidelberg, Germany

**Keywords:** Breast neoplasms, Attendance, Exercise intervention, Neoadjuvant chemotherapy

## Abstract

**Background:**

Performing 2–3 exercise sessions/week may relieve therapy-related side effects of breast cancer patients (BRCA) and improve their quality of life. However, attendance to the exercise sessions is often impaired. Thus, we investigated patterns and possible influencing factors of attendance to an aerobic (AT) or resistance training (RT) intervention in BRCA during neoadjuvant chemotherapy.

**Methods:**

BRCA (*N* = 122) were randomly allocated to supervised AT or RT twice weekly during neoadjuvant chemotherapy (18 ± 4 weeks). Attendance was calculated individually and group-wise per training week as the percentage of the performed sessions out of the prescribed sessions. Possible influencing factors were investigated using multiple regression analyses.

**Results:**

Mean individual attendance was 44.1% ± 29.3% with no significant differences between the groups. Group-wise attendance was highest in the first 6 weeks of training with ≥ 60% for AT and ≥ 50% for RT, but decreased over the course of the intervention accompanying chemotherapy. Significantly higher attendance was associated with not having vs. having nausea (*ß* =  − 14.57; *p* = 0.007) and not having vs. having pain (*ß* =  − 12.07; *p* = 0.12), whereas fatigue did not show any association (*ß* =  − 0.006; *p* = 0.96). Having been randomized into a preferred intervention group (48.8%) showed no association with attendance. Yet, patients’ rating of the exercise intervention as “good”/ “very good” (58.7%) was significantly associated with higher attendance (*p* = 0.01).

**Conclusion:**

For both exercise interventions, group-wise attendance/training week decreased during chemotherapy despite good intervention ratings. While some patients never started, others trained almost constantly twice weekly. The study revealed that patients who are nauseous or experience pain may need more support to attend more exercise sessions.

**Trial Registration Clinicaltrials.gov**: NCT02999074 from May 6, 2016.

## Introduction

Improved breast cancer therapy leads to continuously increasing number of survivors, but is often accompanied with therapy-related side effects that impede the physical and psychological health and, thus, the daily living of the individual [24]. There is convincing evidence that exercise interventions that comply with the exercise guidelines for cancer survivors according to the American College of Sports Medicine (ACSM) are safe and feasible and were further shown to be associated with significant improvements of fatigue, health-related quality of life, and physical fitness during and after cancer treatment in cancer survivors [5]. These health benefits were achieved with an aerobic or resistance training of moderate intensity that was performed 2–3 times per week over 12 weeks with an intensity of 60–85% of the maximal heart rate for the aerobic and 60–80% of the one-repetition maximum (1-RM) for the resistance training [5]. Thus, in order to improve experienced treatment-related side effects, not just the training specifications (i.e., frequency, intensity, time, and type of the prescribed exercise) are important, but how well they are followed, i.e., the adherence to the training prescriptions.

Adherence is usually defined as following the given recommendations [25], which means for an exercise intervention that the respective exercise sessions are performed as they were prescribed regarding frequency, intensity, time, and type of the prescribed exercise (i.e., the FITT-principles). Yet, it is very complex to assess all these criteria over all sessions, especially during chemotherapy when exercise prescriptions frequently are adjusted to the impaired health status of the patients. Even though there are some approaches to measure adherence through the performed training volume, required dose modifications, and missed progression steps [26], there is no gold standard of measuring the adherence to an exercise intervention. Thus, as a feasible surrogate measure, often attendance to the exercise sessions is assessed [15].

Attendance is defined as participating in an exercise session and performing training irrespective of intensity, duration, or any other exercise prescriptions [14]. Therefore, the overall attendance to an exercise program is usually assessed as the percentage of exercise sessions attended out of the scheduled number of sessions [16, 20, 23]. Attendance of breast cancer patients in exercise programs in the current literature varies between 41 and 83% [10, 13, 23, 27, 31]. Different factors have been associated with attendance such as therapy-related side effects, work or family duties, travel distance, socio-demographics (e.g., education and marital status), lifestyle habits (physical fitness, exercise history, smoking, and alcohol status), psychological determinants (e.g., self-efficacy and mood), and a lack of motivation and/or time [2, 9, 12, 17, 20, 22, 31]. However, current evidence on influencing factors of attendance to an exercise intervention besides the well-known like the marital status or higher education is still inconclusive. Additionally, influencing factors were usually assessed either prior [3, 17, 22, 29, 31], or after completing the cancer therapy [3, 9, 10], or after treatment but prior to exercise intervention [9, 11]. But it may be more meaningful to assess all factors that are susceptible to the cancer treatment during the cancer treatment. Further, it has been insufficiently investigated if and how the exercise attendance varies during chemotherapy. So far, research was mainly conducted in patients after or during adjuvant treatment irrespective of the physical activity behavior, but yet only scarcely in rather physically inactive patients or patients undergoing neoadjuvant treatment.

Therefore, our aims were to investigate (1) the individual attendance of rather physically inactive breast cancer patients to a randomly prescribed supervised exercise intervention during neoadjuvant chemotherapy, (2) group-wise attendance per training week during chemotherapy, and (3) possible influencing factors with regard to socio-demographics, therapy-related side-effects, pre-diagnosis physical activity behavior, and patient’s randomization preferences.

## Methods

We investigated data of a 3-arm randomized controlled exercise intervention trial where breast cancer patients undergoing neoadjuvant chemotherapy had been randomized to an aerobic (AT) or resistance exercise training (RT) during neoadjuvant chemotherapy or to a usual care/waitlist control group (UC), who received resistance exercise after breast surgery (BENEFIT study, clincialtrials.gov NCT02999074). As we aimed to investigate adherence to exercise interventions during neoadjuvant chemotherapy, we did not consider the UC group in the present analyses.

The BENEFIT study was approved in 2015 by the ethics committee of the Medical Faculty of Heidelberg and the recruitment took place from January 2016 to October 2022.

**Fig. 1:**
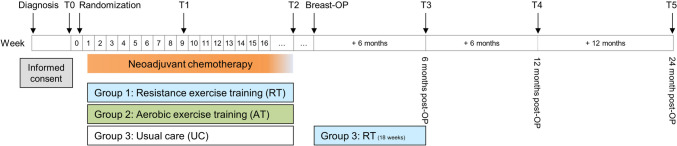
BENEFIT study scheme. In the present analyses, we considered adherence to exercise interventions during neoadjuvant chemotherapy. Thus, we did not consider group 3 (UC) here

The computerized block randomization to study arms (1:1:1) was stratified by the tumor type (HR − , HR + /HER2 + , and HR + /HER2 −) and performed after completion of the first study assessment (baseline, T0) prior to the first chemotherapy (Fig. 1). Further assessments took place 9 weeks after therapy start (T1), after completing the chemotherapy (T2), and 6 (T3), 12 (T4), and 24 months (T5) after breast surgery.

### Participants

Eligible study participants were females ≥ 18 years old with a histologically confirmed primary breast carcinoma, a body mass index of ≥ 18 kg/m^2^, scheduled for neoadjuvant chemotherapy (not yet started), sufficient German language skills, willing to participate in the study measurements at our study center, and exercise twice weekly in one of our cooperating exercise facilities. Already systematically exercising patients (i.e., exercising at least 2 × 1 h/week) or having any health condition that might hamper the study participation were not included.

### Interventions

According to the ACSM exercise guidelines, a supervised training was performed twice weekly in a training facility and a 15-min home-based exercise training without supervision once weekly.

Patients randomized to AT started with a 6-week continuous training at 60% of their VO_2_max (15–30 min per training session) that progressed to 70% VO_2_max (30–60 min per training session). From the seventh week onwards, an interval training was introduced consisting of four intervals at 75–85% VO_2_max with a responsively recreational interval at 60% VO_2_max. The machine on which the endurance training was performed could be individually chosen for every training session, but most patients performed it on a bicycle ergometer and only a few patients chose a rowing machine, treadmill, or elliptical trainer.

Patients randomized to RT received a machine-based strength training, comprising all major upper and lower muscle groups (leg extension, leg curl, leg press, shoulder internal and external rotation, seated row, latissimus pull down, butterfly and butterfly reverse). Each exercise consisted of 3 sets with 8–12 repetitions each and a training weight corresponding to 60–80% of the individual’s 1-RM (performed after two familiarization sessions) [1, 6, 28].

Patients randomized to UC did not receive any kind of exercise intervention or specific recommendations during the neoadjuvant chemotherapy but received the same machine-based strength training than RT after their surgery and with medical clearance only.

### Measures

We considered both, the individual and group-wise attendance per training week. In accordance with the literature, we calculated the individual’s attendance as the number of attended supervised exercise sessions divided by the number of prescribed supervised exercise sessions, multiplied by 100 [16, 20, 23]. Prescribed were two supervised exercise sessions weekly over a period starting within one week after the admission of the first chemotherapy (or in a few cases with slight delays due to administrative issues) until the T2 study assessment (after the end of the chemotherapy). As the duration of the chemotherapy and exercise period may vary between the patients, the amount of prescribed supervised exercise sessions and the individual attendance was separately calculated for each patient. Additionally, we considered the group-wise attendance per training week to assess the variation during the intervention and chemotherapy. For this purpose, the actual numbers of performed supervised exercise sessions per week were added up cumulatively over all patients in that group, thereafter divided by the number of expected sessions for that group, and multiplied by 100.

The attendance to the unsupervised home-based training was not considered in the analyses.

### Assessment of attended supervised exercise sessions

The attendance to a session was documented by the signature of the training facility, and additionally through case-report forms completed by the patient prior and after each exercise session (to assess the safety of the intervention). Additionally, the patients were called every other week to ensure a healthy and safe training and for keeping up the motivation and attendance. These phone calls were also documented.

If a patient never started the training, the attendance was set to zero.

### Assessment of patient-related factors

The socio-demographics, previous experience with resistance training (yes/no), and the physical activity behavior in the youth 12 months prior to the study were assessed through a questionnaire completed by the patients at baseline. The physical activity behavior was differentiated into walking for more than 20 min at once, riding a bike, e.g., transportation or alike, but not for exercise reasons for more than 20 min at once and every kind of sport they performed. Additionally, the patients reported which kind of activity they performed on how many months within the previous 12 months prior study start, the number of active days per month, the duration they spent in the activity in minutes per active day, and the exercise intensity (low, moderate, partially vigorous, mostly vigorous).

Height and weight were measured by study personnel at baseline and used to calculate the BMI as kg/m^2^. The age was extracted from the patient’s medical record.

The travel distance from each patient’s home to her training facility was extracted with Google Maps. The shortest distance by car was taken.

Exercise preference at baseline was assessed after the intervention period, with a self-developed questionnaire. The patients could choose between the given response options “AT during NACT,” “RT during NACT,” “any training during NACT,” “RT after surgery,” and “no preferences.” The responses were categorized according to their randomized arm into having received the preference (yes/no).

### Patient-reported outcomes

The patient-reported outcomes with regard to quality of life, fatigue, and chemotherapy-related side effects were assessed using the standardized European Organization for Research and Treatment of Cancer questionnaire modules (i.e., EORTC QLQ-C30, -CIPN20, -FA12). Depression and anxiety were assessed with the short Patient Health Questionnaire (PHQ-4) and perceived social support via the Multidimensional Scale of Social Support (MSPSS).

As chemotherapy is known to impede the patients’ physical and psychological health, which may influence exercise attendance, the patient-reported outcomes assessed at T1 were used for the analyses.

### Statistical methods

The impact of the aforementioned parameters on the individual exercise attendance was investigated with multiple linear regression analysis. Based on literature or theoretical considerations on influencing factors, the model included the randomized group (AT/RT), age, BMI, being married/living with a partner (yes/no), education (university degree/high school/lower), and physical activity behavior 12 months prior study start in the areas walking, cycling and sports (log-transformed). Additionally, the model simultaneously included potential side effects of cancer therapy, i.e., fatigue (continuous), pain (none/mild/medium/severe), and nausea (present/not present). To avoid overfitting, all other variables, such as experience with resistance training, exercise in the youth, chemotherapy-induced peripheral neuropathy, anxiety and depression, the perceived social support, being currently employed, having underaged children, the travel distance to the training facility (log-transformed) and having received the preferred exercise intervention, were separately added to the abovementioned model to investigate its influence on the exercise attendance. The fit diagnostics panels and variance inflation factors indicated no conflict with the regression assumptions.

All statistical analyses were conducted with SAS (version 9.4), and all tests were performed two-sided with *p* < 0.05 considered as statistically significant.

## Results

### Study population

Of all patients scheduled for an appointment at the NCT, 952 patients were identified as potentially eligible, of which 262 had to be excluded due to the in-/exclusion criteria. Of 185 patients who agreed to participate, all patients who were randomized in AT (*N* = 61) or RT (*N* = 61) during chemotherapy finished the training and, thus, were included in the analyses. The baseline characteristics are presented in Table 1.

**Table 1:** Patient characteristics

Variable	AT (*N* = 61)		RT (*N* = 61)		Two-sided *p*-value
Age, mean (SD)	51.70	11.40	48.60	10.70	0.13
Body mass index, mean (SD)	25.70	5.90	25.60	4.10	0.94
Marital status, *N* (%)					
Married/living with a partner	47.00	77.10	47.00	79.70	0.73
Living alone	14.00	23.00	12.00	20.30	
Having children < 18 years, *N* (%)	19.00	31.20	25.00	42.40	0.21
Currently employed, *N* (%)	20.00	32.80	19.00	31.70	0.90
Education, *N* (%)					
University degree	25.00	41.00	24.00	40.00	0.70
High school graduation	13.00	21.70	8.00	13.30	
Lower	23.00	38.30	28.00	46.70	
Experience with resistance training, *N* (%)	33.00	54.10	32.00	54.20	0.99
Exercise in youth, *N* (%)	44.00	72.10	43.00	70.50	0.84
Physical activity behaviour [median MET-h/week, Q1–Q3] 12 months prior study					
Walking	4.30	1.6–10.4	7.40	3.2–15.0	0.04
Cycling	0.70	0.0–4.0	0.90	0.0–6.1	0.78
Sports	0.90	0.0–3.9	1.20	0.0–4.5	0.84
Fatigue, mean (SD)	32.50	24.00	33.90	21.40	0.72
Pain					
No	12.00	20.00	25.00	41.00	0.94
Mild	16.00	26.70	10.00	16.40	
Moderate	13.00	21.70	6.00	9.80	
Severe	16.00	26.70	16.00	26.20	
Nausea	16.40	18.20	14.60	19.70	0.62
Perceived social support, mean (SD)	93.70	15.40	91.40	13.80	0.40
Neoadjuvant chemotherapy					
A + C + T + P	11.00	47.80	12.00	52.20	0.72
A + C + T	28.00	44.40	35.00	55.60	0.38
A + C	32.00	46.40	37.00	53.60	0.28
T + P	30.00	50.80	29.00	49.20	0.72
C + T	32.00	43.80	41.00	56.20	0.38
Anti-Her2 antibodies	16.00	13.60	17.00	14.40	0.67
Other antibodies	7.00	5.90	4.00	3.40	0.41
Missing data	1.00	0.80	2.00	1.60	0.55

*A*, anthracyclines; *AT*, aerobic exercise training; *C*, cyclophosphamides; *MET-h/week*, metabolic equivalent of task in hours per week; *P*, platin derivates; *RT*, resistance exercise training; *T*, taxanes; *Q1*, first quartile; *Q3*, third quartile

The average age was 50.1 (± 11.1) years, and the average BMI was 25.6 (± 5.1) kg/m^2^. The population was well educated with 57.4% having high school or university degree. About a third of participants (32.2%) were still working at baseline despite their cancer diagnosis, of whom the majority (43.6%) had a lower educational background.

Most of the participants were married/living with a partner (78.3%) and had one (25.0%) or two children (39.2%), who were mostly above the age of 18 years (63.3%). All patients were rather physically inactive within the 12-month prior baseline. The median physical activity of the RT group was higher in walking, cycling, and sport than of the AT group, but reached statistical significance only for walking (*p* = 0.04).

### Attendance to the prescribed exercise sessions

#### Individual attendance

The exercise intervention had a mean (± SD) duration of 21 (± 4) weeks. Of all, 22 patients (RT: 8, AT: 10) had an intervention duration above 23 weeks.

**Fig. 2:**
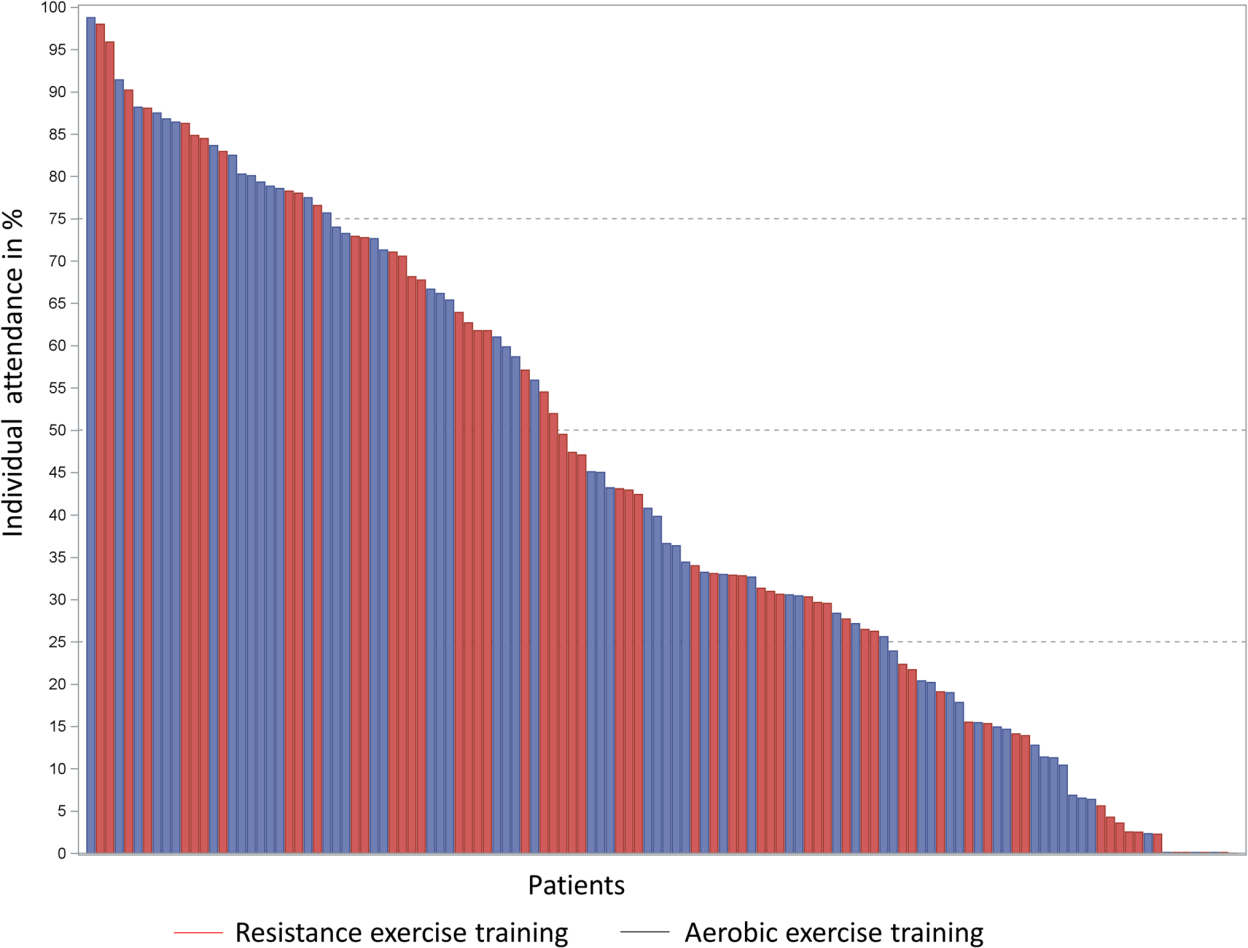
Individual attendance in the two training groups. For each patient, the attendance is represented by a separate vertical bar, sorted by attendance

Overall, seven patients (5.7%; RT: 4, AT: 3) never started their training due to cancer therapy-related side effects or psychological issues. The overall mean (± SD) attendance was 44.1% (± 29.3%) with no significant differences between the groups (RT: 43.0% (± 29.1%), AT: 45.2% (± 29.7%), *p* = 0.07). The individual attendance is displayed in Fig. 2 with each vertical bar representing the attendance to the prescribed exercise sessions of a single patient, sorted by attendance. Of all patients, 65 attended < 50% and 50 patients ≥ 50% of the supervised exercise sessions.

#### Group-wise attendance per training week

**Fig. 3:**
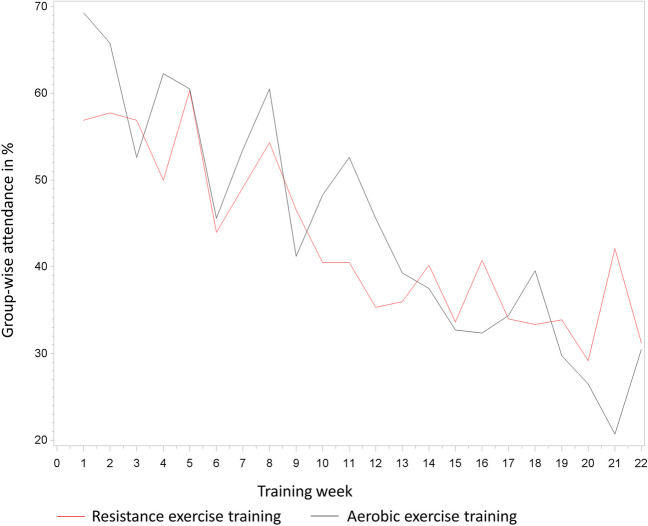
Group-wise attendance per training week

Besides the individual attendance to the supervised exercise, the group attendance per training week (Fig. 3) was investigated.

According to the study protocol, the patients should attend two exercise sessions per week, but as some patients started their training delayed (*N* = 3) or even never (*N* = 7) and some did not manage to come to the second session, the attendance is already below 100% in the first week of training. The number of patients, who attended both exercise sessions, varied between 2 and 35% during the intervention phase. A continuous decrease of patients attending the exercise sessions twice weekly from week 6 was observed (data not shown).

The average group-wise attendance per training week was 41.8% (± 12.1%) with no significant differences between the groups (RT: 40.3% (± 9.4%), AT: 43.4% (± 14.3%)).

### Factors influencing the individual exercise attendance

**Fig. 4:**
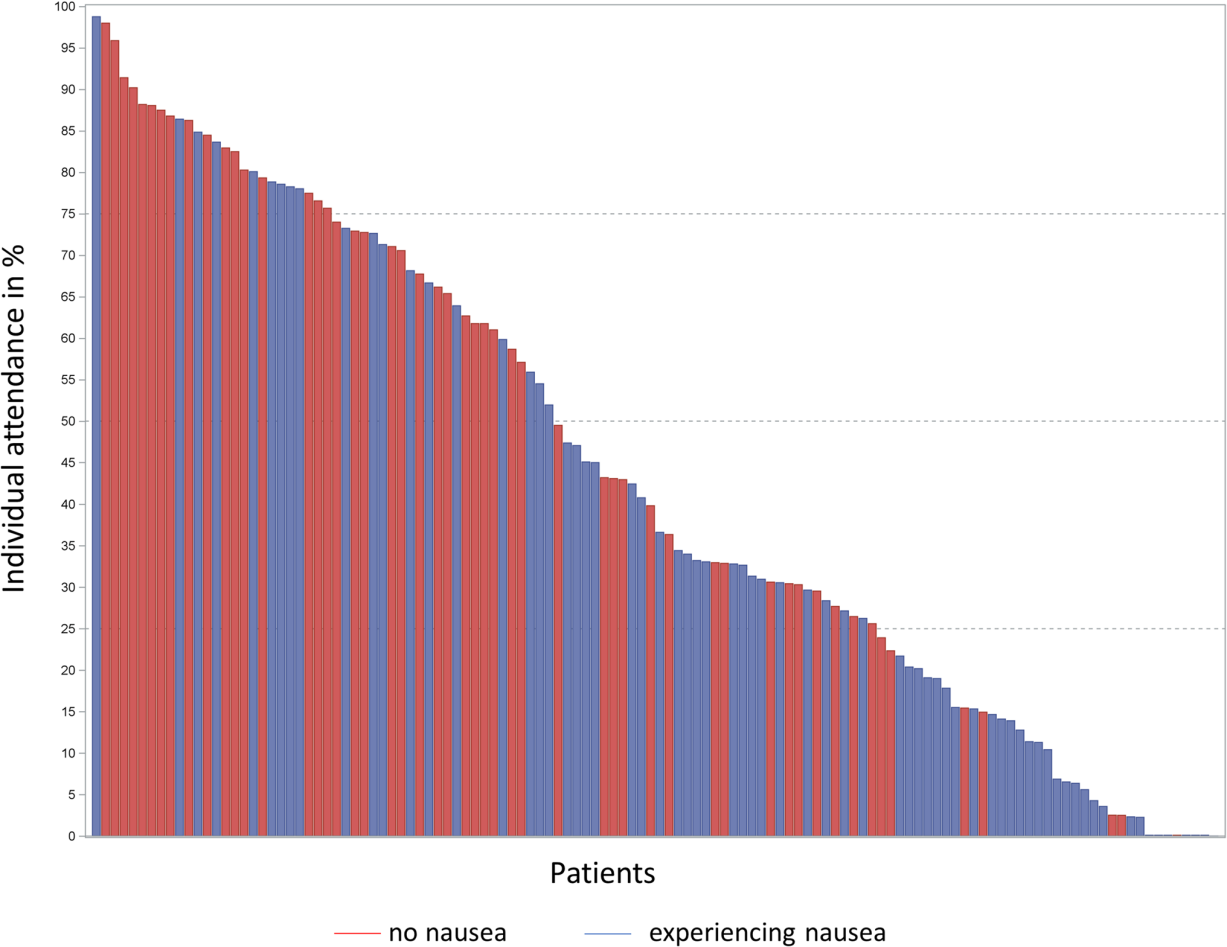
Individual attendance to the training by experiencing nausea

Significantly lower attendance was observed for a higher amount of walking prior study start (*ß* =  − 6.21; p = 0.006), higher BMI (*ß* =  − 1.68; *p* = 0.0007), living alone (*ß* =  − 16.35; *p* = 0.007), high school graduation (*ß* =  − 23.59; *p* = 0.0005), having nausea (*ß* =  − 14.57; *p* = 0.007; Fig. 4) or rated the exercise intervention as “very poor”/ “poor”/ “OK” (*ß* =  − 21.78; *p* = 0.004). All other investigated factors neither showed a significant association with attendance nor any confounding effect on the other variables in the model (Table 2).

**Table 2:** Determinants of individual exercise attendance

Variable	Beta-estimate	Standard error	Two-sided *p*-value
Group			
Aerobic exercise training		Reference	
Resistance exercise training	− 1.50	4.75	0.75
Age	0.31	0.21	0.14
Body mass index	− 1.68	0.48	0.0007
Marital status			
Married/living with a partner		Reference	
Living alone	− 16.35	5.92	0.007
Education			
University degree		Reference	
High school graduation	− 23.59	6.56	0.0005
Lower	− 0.51	5.17	0.92
Fatigue	− 0.006	0.12	0.96
Pain			
No		Reference	
Mild	1.96	6.58	0.77
Moderate	− 3.57	7.28	0.63
Severe	− 12.07	7.71	0.12
Nausea			
No		Reference	
Yes	− 14.57	5.26	0.007
Physical activity behaviour			
Walking	− 6.21	2.22	0.006
Cycling	− 2.10	2.03	0.30
Sports	2.41	2.03	0.24
Patients’ rating of the exercise			
“Good” or “very good”		Reference	
“Very poor,” “poor,” and “OK”	− 21.78	7.33	0.004

The exercise preference prior study start was only available for 91 patients (74.6%). Of these, 8 (8.8%) had no preferences regarding the randomization, 9 (9.9%) preferred to exercise after the surgery and the remaining participants preferred RT (*N* = 25, 27.5%), AT (*N* = 25, 27.5%), or any training (*N* = 24, 26.4%) during NACT. Overall, the randomization matched the preferences of 63 patients (51.6%). Having received the preferred exercise intervention was not associated with a higher exercise attendance (*ß* = 6.91; *p* = 0.24).

To consider the influence of the COVID-19 pandemic and the related restrictions in Germany from March/April 2020 until spring 2023, the attendance of the patients exercising before and after March/April 2020 was compared. The patients who exercised prior to the COVID-19 pandemic had a slightly higher attendance (median: 45.0%; Q1: 21.7%, Q3: 73.3%) than those who were affected by the COVID-19 pandemic (median: 33.1%; Q1: 19.0%, Q3: 66.7%). These differences were statistically not significant (*p* = 0.38).

## Discussion

In this study, we investigated (1) the attendance to an aerobic and resistance exercise intervention in 122 breast cancer patients that was performed during neoadjuvant chemotherapy and (2) possible influencing factors that were assessed pre- and mid-intervention.

The overall attendance was rather low with 59% of the patients attending below 50% of all prescribed sessions, including seven patients who never started training due to too severe side effects of the chemotherapy.

The mean attendance of 44% (± 29%) in our study was comparable to the attendance observed during chemotherapy weeks of Kirkham and colleagues (2020), when the patients received a standard linear exercise prescription (57% (± 30%)) compared to chemotherapy-periodized exercise prescription (77% (± 28%)) [19]. The chemotherapy-periodized exercise prescription was associated with a statistically significantly higher exercise attendance (*p* = 0.05) [19]. Although the patients in our study had the opportunity to reduce the intensity of their exercise program if they perceived it as too intense, it cannot be excluded that a chemotherapy-periodized exercise prescription according to the experienced side effects might have increased the attendance to some extent. This may be a good approach for future studies.

Yet, the observed attendance in our study was also lower than the reported average attendance in other exercise intervention studies with breast cancer patients undergoing chemotherapy, ranging from 63 to 82% [3, 4, 9, 16, 22, 30]. One possible explanation might be potential differences in chemotherapy regimens. About two-thirds of our patients received at least in part weekly chemotherapy regimen, which might have led to more severe side-effects and thus, lower exercise attendance. Thereby, the length of the chemotherapy may be of interest too, as it appears to be intuitive that side effects accumulate over time. Previous studies observed a lower attendance with a longer chemotherapy protocol [9, 20, 22]. Chemotherapy-related side effects, along with time issues due to the appointment schedule of the training facilities or medical appointments, were reported by patients in previous studies, who were not able to attend their exercise sessions as prescribed [2, 13, 19, 21, 30]. Yet, in our multiple regression analyses, regarding chemotherapy side effects, only nausea had a statistically significant negative influence on the exercise attendance. Pain tended to have a negative association with attendance (*ß* =  − 12.07; *p* = 0.12). Patients also reported pain as a reason to skip exercise sessions in the biweekly adherence calls by our study personnel in line with previous studies [2, 8, 21]. Interestingly, fatigue did not appear to influence the attendance at either exercise intervention, which is in line with some [9], but not all studies [2, 19, 21, 31]. These results may be drawn back to their assessment time, as we assessed the variables mid-intervention, whereas previous studies investigated possible influencing factors mostly either prior [3, 9, 11, 17, 22, 29, 31] or after completing the cancer therapy [3, 9, 10], which may have led to a misinterpretation of the association between the treatment-related side-effects and exercise attendance. Another possible explanation for the lower attendance may be the restriction of the study population to rather physically inactive patients. An association between the PA behavior prior to study entry and attendance at exercise intervention was observed [3, 18, 29], and previous studies tended to include patients irrespective of their activity level [10, 22, 31].

The COVID-19 pandemic might have additionally contributed to the rather low exercise attendance, with the intervention of 57 patients (46.7%) having been after the onset of COVID. These patients might have been more reluctant to go to a public gym due to fears of infection. Additionally, it had been required to wear a medical face mask from the end of September 2020 until Spring 2023 to prevent a COVID-19 infection, which may have made exercising less pleasant. This supposed effect of the COVID-19 pandemic is in line with our data showing a somewhat lower median attendance among the patients who received the intervention compared to those who exercised prior COVID. Otherwise, the COVID-19 pandemic might have also led to a selection bias, in the way that the patients who participated despite pandemic-related obstacles may have been generally more motivated or having a less anxious personality.

In line with previous studies, we observed that patients with a lower BMI [9, 13, 22, 31] or a university degree [13, 31] had a statistically significantly higher exercise attendance, but not the type of exercise (AT/RT) [10]. This is in line with the study of Courneya and colleagues (2009) [7]. Our results on the comparison of AT versus RT can hardly be discussed in the context of previous studies, as they barely investigated differences between AT and RT. Also, we did not observe an association between receiving the preferred intervention and attendance.

Patients, who were married/living with a partner, attended significantly more exercise sessions than unpartnered patients. This may be attributed to the social and/or practical support with family duties, including taking care of the children and the household or driving the patient to the training facility, as also suggested by others [30]. Interestingly, the perceived social support was not associated with the exercise attendance. Additionally, the travel distance to the training facility needs to be considered as this may hinder patients to regularly attend their prescribed exercise regimen. In contrast to studies of Courneya and colleagues (2008; 2014), we did not observe an association between the travel distance and the exercise attendance [9, 10]. We suggest that this is attributable to the chosen training facility that was as close to the patient’s home as possible and discussed with each patient prior study entry.

Limitations of our study need to be considered, including the investigation of solely the exercise attendance instead of the adherence to the exercise program. Additionally, the retrospective assessment of the exercise preferences may have been misleading as the patient’s choice may have been influenced by the received intervention as well as the experienced chemotherapy-related side effects, as spontaneously reported by a few patients.

Furthermore, personality, self-efficacy, and perceptions of the patients were not assessed but might play a role regarding exercise attendance.

Strengths of our study encompass the balanced number of patients in AT and RT that enabled group comparisons, the comparison of two different types of exercises, and the low baseline activity level of the patients. The exercise attendance was assessed using case report forms filled in by the patient and a list of signatures in the training facility that reduced the risk of overreporting. Through the assessment of the chemotherapy-related side effects mid-intervention, the increasing influence of the chemotherapy on the attendance at exercise could be best assessed and was reflected in a continuous decrease of the attendance during treatment as was observed in a previous study [2].

## Conclusion

Even though most patients rated the exercise intervention as “good” or “very good” and were continuously encouraged to exercise, if necessary with reduced intensity, the group-wise attendance decreased during chemotherapy treatment and was on average rather low. Yet, there was large heterogeneity with some patients never starting, whereas others trained twice weekly almost throughout the whole chemotherapy period.

The absence of nausea and pain was associated with higher exercise attendance, whereas fatigue showed no such association.

## Data Availability

The data that support the findings of this study are available from the corresponding author upon reasonable request.
